# Porphyrin biosynthesis in human Barrett's oesophagus and adenocarcinoma after ingestion of 5-aminolaevulinic acid

**DOI:** 10.1054/bjoc.2000.1300

**Published:** 2000-07-24

**Authors:** P Hinnen, F W M de Rooij, E M Terlouw, A Edixhoven, H van Dekken, R van Hillegersberg, H W Tilanus, J H P Wilson, P D Siersema

**Affiliations:** Departments of Gastroenterology and Internal Medicine II, Pathology, Surgery, University Hospital Rotterdam-Dijkzigt, Dr. Molewaterplein 40, Rotterdam, GD 3015, The Netherlands

**Keywords:** photodynamic therapy, 5-aminolaevulinic acid, Barrett’s oesophagus, porphyrin biosynthesis

## Abstract

5-Aminolaevulinic acid (ALA)-induced porphyrin biosynthesis, which is used for ALA-based photodynamic therapy (ALA-PDT), was studied in tissues of 10 patients with Barrett’s oesophagus (BE) and adenocarcinoma of the oesophagus (AC) undergoing oesophagectomy at a mean time interval of 6.7 h after the ingestion of ALA (60 mg kg^–1^). In BE, AC, squamous epithelium (SQ) and gastric cardia, the activities of the haem biosynthetic enzymes porphobilinogen deaminase (PBG-D) and ferrochelatase (FC) and the PDT power index – the ratio between PBG-D and FC in BE and AC in comparison with SQ – were determined before ALA ingestion. Following ALA administration, ALA, porphobilinogen, uroporphyrin I and PPIX were determined in tissues and plasma. The PDT power index did not predict the level of intracellular accumulation of PPIX found at 6.7 h. In BE, there was no selectivity of PPIX accumulation compared to SQ, whereas in half of patients with AC selectivity was found. Higher haem biosynthetic enzyme activities (i.e. PBG-D) and lower PPIX precursor concentrations were found in BE and AC compared to SQ. It is therefore possible that PPIX levels will peak at earlier time intervals in BE and AC compared to SQ. © 2000 Cancer Research Campaign


*British Journal of Cancer* (2000) **83**(4), 539–543
© 2000 Cancer Research Campaign
doi: 10.1054/ bjoc.2000.1300, available online at http://www.idealibrary.com on IDEAL®

### Porphyrin biosynthesis in human Barrett's oesophagus and adenocarcinoma after ingestion of 5-aminolaevulinic acid

**P Hinnen[1], FWM de Rooij[1], EM Terlouw[1], A Edixhoven[1], H van Dekken[2], R van Hillegersberg[3], HW Tilanus[3], JHP Wilson[1] and PD Siersema[1]**

Departments of [1]Gastroenterology and Internal Medicine II, [2]Pathology and [3]Surgery, University Hospital Rotterdam-Dijkzigt, Dr. Molewaterplein 40, 3015 GD Rotterdam, The Netherlands

**Summary** 5-Aminolaevulinic acid (ALA)-induced porphyrin biosynthesis, which is used for ALA-based photodynamic therapy (ALA-PDT), was studied in tissues of 10 patients with Barrett's oesophagus (BE) and adenocarcinoma of the oesophagus (AC) undergoing oesophagectomy at a mean time interval of 6.7 h after the ingestion of ALA (60 mg $kg^{-1}$). In BE, AC, squamous epithelium (SQ) and gastric cardia, the activities of the haem biosynthetic enzymes porphobilinogen deaminase (PBG-D) and ferrochelatase (FC) and the PDT power index – the ratio between PBG-D and FC in BE and AC in comparison with SQ – were determined before ALA ingestion. Following ALA administration, ALA, porphobilinogen, uroporphyrin I and PPIX were determined in tissues and plasma. The PDT power index did not predict the level of intracellular accumulation of PPIX found at 6.7 h. In BE, there was no selectivity of PPIX accumulation compared to SQ, whereas in half of patients with AC selectivity was found. Higher haem biosynthetic enzyme activities (i.e. PBG-D) and lower PPIX precursor concentrations were found in BE and AC compared to SQ. It is therefore possible that PPIX levels will peak at earlier time intervals in BE and AC compared to SQ. © 2000 Cancer Research Campaign

**Keywords:** photodynamic therapy; 5-aminolaevulinic acid; Barrett's oesophagus; porphyrin biosynthesis

Barrett's oesophagus (BE) is a pre-malignant condition in which progression from metaplasia to low-grade dysplasia and high-grade dysplasia could lead to invasive adenocarcinoma of the oesophagus (AC) (Hameeteman et al, 1989; van der Burgh et al, 1996; Drewitz et al, 1997). High-grade dysplasia is often regarded as an indication for oesophagectomy (Clark et al, 1996; Edwards et al, 1996; Cameron and Carpenter, 1997). A possible alternative, which is less mutilating and also applicable in patients with a high surgical risk, is 5-aminolaevulinic acid-induced photodynamic therapy (ALA-PDT).

Two relevant clinical studies have been performed, in which patients with high-grade dysplasia or early cancer in BE received an oral dose of ALA (60 mg $kg^{-1}$), followed by photoactivation 4–6 h later (Barr et al, 1996; Gossner et al, 1998). Both high-grade dysplasia and early cancer were eradicated allowing regeneration of squamous epithelium without scarring or stricture formation. However, the presence of islands of columnar cells remaining beneath regenerating squamous epithelium created the concern that superficial healing could mask underlying dysplasia. These results suggest that ALA-PDT needs to be improved.

Haem biosynthesis, an essential process in every cell, is the basis of ALA-PDT (Figure 1). ALA is the first intermediate, and two molecules of ALA are converted to porphobilinogen (PBG) which is metabolized to porphyrinogen intermediates by porphobilinogen deaminase (PBG-D). The last step of haem biosynthesis is the insertion of iron into PPIX by ferrochelatase (FC). Normally, haem synthesis is regulated by feedback inhibition of the enzyme ALA synthase. Exogenous ALA bypasses this feedback inhibition and the activities of PBG-D and FC and the intracellular iron pool become rate-limiting factors. As a result porphyrins, predominantly PPIX, will accumulate (Bishop and Desnick, 1982; Kennedy and Pottier, 1992). Previously, we observed an imbalance between the activities of PBG-D and FC in BE and AC (Hinnen et al, 1998). The ratio between PBG-D and FC activities, normalized for squamous epithelium, was found to be significantly higher in BE and AC. In that study, we suggested that this ratio, which we have called the PDT power index, might be a useful parameter for predicting the accumulation of PPIX in tissues after the administration of ALA.

In this study, we examined the relation between the PDT power index and the intracellular concentration of PPIX in tissues of patients with BE and AC at approximately 6 h after ALA ingestion (60 mg $kg^{-1}$) as this is the clinically most frequently used time interval. We determined the intracellular concentrations of ALA and other haem intermediates by biochemical extraction methods rather than fluorescence microscopy as used by others (Regula et al, 1995; Barr et al, 1996). In addition, plasma pharmacokinetics of ALA and porphyrins were studied and side-effects were monitored.

*Received 11 January 2000*
*Revised 19 February 2000*
*Accepted 17 April 2000*

*Correspondence to:* FWM de Rooij

### MATERIALS AND METHODS

### Patients

In total 10 patients (two women and eight men; age 44–81 years; mean 65 years) gave their written informed consent to participate in this study, which was approved by the Medical Ethical

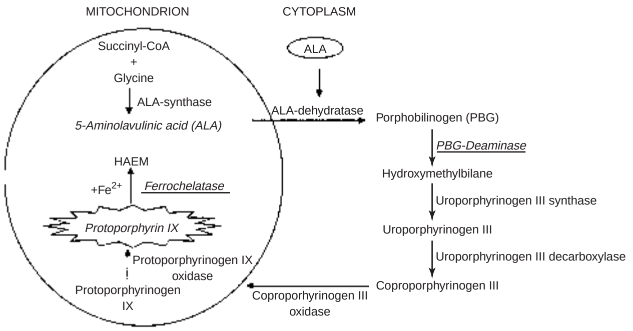

**Figure 1** Haem biosynthetic pathway

Committee of the University Hospital Rotterdam. Nine patients with histologically proven AC in BE and one patient with high-grade dysplasia in BE underwent an oesophageal resection with a gastric tube interposition. One patient was excluded from analysis because the tissue samples taken from BE were contaminated with AC as samples were taken at the border between BE and AC.

### Study design

### *Biopsy samples (pre-5-aminolaevulinic acid administration)*

Apart from one patient, all patients underwent an endoscopy with biopsies taken from BE, AG normal gastric cardia mucosa (GC) and normal squamous epithelium (SQ). Biopsies were embedded in formalin, sectioned, and stained with haematoxylin and eosin. The grade of tumour differentiation and the grade of dysplasia in Barrett's mucosa were described according to Haggitt (Haggitt, 1994). In addition, adjacent biopsies were kept at –70°C until the activities of PBG-D and FC and porphyrin concentrations were determined (Hinnen et al, 1998).

### *5-Aminolaevulinic acid administration*

Six hours before the oesophageal resection, ALA (Fluka, Buchs, Switzerland, 60 mg $kg^{-1}$) was dissolved in orange juice (10 ml, at room temperature) and given to the patient. Following this, all patients drank an additional 30 ml of water.

Photodegradation of porphyrins and photosensitization tissue-damage during exposure to the operating lights was prevented by covering the tissues not in the immediate operating field with gauzes and shielding the operating lights with acrylate yellow filters (Wientjes BV, Roden, The Netherlands), which eliminate nearly all UV and blue light below a wavelength of about 520 nm (Hinnen et al, 2000*a*). For 48 h after the administration of ALA, patients were kept in subdued light. Side-effects were monitored by questionaires and physical examination.

### *Blood samples*

Venous blood samples were collected prior to and at 1, 3, 6, 9, 12, 24 and 48 h after the administration of ALA. Whole blood was collected in tubes wrapped in aluminum foil to prevent photoconversion and photodamage, and kept on ice. The blood samples were centrifuged at 1300 ***g*** for 10 min, then the plasma was removed, protected from light and stored at –70°C until the determinations of ALA, porphobilinogen (PBG), uroporphyrin (URO) and PPIX. In addition, samples were collected for routine biochemistry (urea, creatinine, sodium, potassium, albumin, alkaline phosphatase, bilirubin, aspartate aminotransferase (ASAT) and alanine aminotransferase (ALAT)).

### *Tissue samples (post-5-aminolaevulinic acid administration)*

Immediately after the oesophageal resection, tissue samples were taken from BE, AC, SQ and GC for histological examination. Adjacent tissue samples were kept at –70°C until the determinations of ALA, PBG, URO and PPIX. It was not always possible to take tissue samples at exactly 6 h after the administration of ALA as in some patients the start of the operation was delayed by the prolonged anaesthetic preparations and in others the anaesthetic procedure was complicated by hypotension (see Results). However six of nine patients were sampled at about 6 ± 0.5 h.

### Laboratory assays

### *Chemicals*

PPIX disodium salt, Zinc-PPIX and PBG were obtained from Porphyrin Products (Logan, UT, USA). Coproporphyrin, URO and Triton X-100 were obtained from Sigma Chemical Co. (St. Louis, MO, USA). Tris-HCL was obtained from Boehringer Mannheim (Mannheim, Germany) and all other chemicals were obtained from Merck (Darmstadt, Germany).

### *Porphobilinogen deaminase and ferrochelatase assays*

Tissue samples, kept on ice, were homogenized in water (1:5, wt/wt) using a Potter Elvehjem homogenizer (Kontess Glass Co., Vineland, NJ, USA). PBG-D and FC activities as well as the PDT power index – the ratio between PBG-D and FC in BE and AC in comparison with SQ – were determined as described previously (Hinnen et al, 1998). Data were expressed as pmol per mg protein per hour. Protein was determined according to the method of Lowry et al (1951).

### *Determinations of 5-aminolaevulinic acid, porphobilinogen, uroporphyrin and protoporphyrin IX in plasma and tissue*

The analysis of ALA and PBG was performed as described previously (van den Boogert et al, 1998). URO was extracted from 25 µl tissue homogenate or plasma (two-fold diluted in NaCl (150 mmol $l^{-1}$) by addition of 200 µl of URO extraction buffer (UEB; Tris-HCl 50 mmol $l^{-1}$, pH 8.0; trichloroacetic acid, 1.5 mol $l^{-1}$ in aqua dest., (3:5, v/v)). After 5 min exposure to UV light (350 nm), to convert porphyrinogens into porphyrins, the samples were centrifuged for 7 min at 3000 ***g***.

The fluorescence of the supernatant was measured at an excitation wavelength of 410 nm and an emission wavelength of 656 nm using a LS 50B spectrofluorometer with a red sensitive photomultiplier (Perkin Elmer, Nieuwerkerk a/d ijssel, The Netherlands). Values were calculated according to a standard curve of URO I in UEB.

Recovery of porphyrins during the extraction was determined by adding standard URO to the samples and in this study recoveries were found in the range of 85–100%. PPIX was extracted from tissue by adding 50 µl PPIX extraction buffer (PEB; Tris-HCl 50 mmol $l^{-1}$, pH 8.0; 425 µl dimethylsulfoxide/methanol, (DMSO/MeOH, 30:70, v/v)) to 25 µl tissue homogenate. The diluted homogenate was mixed vigorously using a vortex and left for about 30 min at room temperature. Samples were then centrifuged for 10 min at 300 ***g***. 100 µl of supernatant was injected on a HPLC as described previously (van Hillegersberg et al, 1992),

 *© 2000 Cancer Research Campaign*

however using an excitation wavelength of 415 nm and an emission wavelength of 630 nm. For the extraction of PPIX from plasma, 950 µl of PEB was added to 50 µl plasma. Values were calculated according to standard curves of Zinc-PPIX and PPIX in DMSO/MeOH (30:70, v/v). Recovery of porphyrins during the extraction was determined by adding standard Zinc-PPIX and PPIX to the samples and in this study recoveries were found in the range of 90–100%. Plasma levels were expressed in nmol $l^{-1}$ and tissue levels in pmol $mg^{-1}$ protein.

### Protoporphyrin IX and its precursors

At 6 h after ALA administration, tissues not only contained PPIX but also other haem synthesis intermediates, which are the precursors of PPIX and therefore considered as potential PPIX. Since the concentration of URO was very low in tissue and plasma samples compared to PPIX, the URO data were omitted from further analysis. PBG is formed from two molecules of ALA and four molecules of PBG form a PPIX molecule. To calculate the potential PPIX molecules present at 6 h after ALA administration, the concentrations of ALA and PBG were divided by 8 and 4 respectively and we called this ‘PPIX equivalents’.

### Statistical analysis

Data are expressed as means ± SEM and were tested for statistical significance using Student’s *t*-test for paired values. Enzyme activities and concentrations of haem intermediates in BE, AC and GC were compared to SQ. Pearson correlation coefficients were calculated to study possible correlations. $P < 0.05$ was considered significant.

### RESULTS

### Porphobilinogen deaminase and ferrochelatase activities and PDT power index

Before oral ALA administration, PBG-D and FC activities were determined in endoscopically derived biopsy samples taken from the oesophagus (BE, AC and SQ) and the proximal stomach (GC). A two-fold increase in PBG-D activity (pmol per mg protein per hr) was found in BE (39.18 ± 5.67, $P$ = 0.013) and in AC (38.76 ± 3.98, $P$ = 0.001) compared with SQ (19.72 ± 2.85), whereas the activity in GC (21.46 ± 1.27) was not different from the activity in SQ (Table 1). The activities of FC (pmol per mg protein per hr) were not significantly different in BE (696 ± 89, $P$ = 0.06) and AC (532 ± 68, $P$ = 0.36) compared to SQ (444 ± 49), whereas the FC activity in GC (688 ± 38, $P$ = 0.02) was significantly increased.

In BE, the PDT power index (1.4 ± 0.2, $P$ = 0.18) was not significantly different from SQ (1.0). In AC, this index was significantly increased (1.9 ± 0.3, $P$ = 0.01) whereas in GC the index was significantly decreased (0.7 ± 0.1, $P$ = 0.01) compared to SQ.

### Protoporphyrin IX and protoporphyrin equivalents concentration in tissue

Tissue samples of nine patients were collected at a mean time interval of 6.7 ± 0.5 h (range 5.25–10) after the administration of ALA. All tissue types contained the same concentrations of ALA-PPIX equivalents (Table 1). In BE, AC and GC, the intracellular concentration of PBG-PPIX equivalents were significantly lower than in SQ.

The individual variability in the concentration of PPIX is demonstrated in Table 2 together with the patients and tissues characteristics. PPIX was the main metabolite of ALA found in tissue. Undetectable low levels of porphyrins were found in tissue samples of any of the nine patients when taken before the oral administration of ALA (results not shown).

The concentration of PPIX in BE (77 ± 17) was not significantly different from SQ (92 ± 15), whereas the concentration in GC was significantly lower (57 ± 10, $P$ = 0.01) (Table 1). Only one patient (Table 1, patient 5) showed a selective accumulation of PPIX in BE compared with SQ. Levels of PPIX did not depend on the grade of dysplasia found in BE.

The concentration of PPIX in AC (112 ± 45) was not significantly different from SQ (92 ± 15) (Table 1). Selective accumulation of PPIX was seen in four cases of AC. Of the remaining four cases of AC, in one patient tissue was obtained at 10 h after the administration of ALA and in the other three cases the AC was histologically found to be poorly differentiated.

The PDT power index did not correlate with the levels of PPIX found.

### Pharmacokinetics of 5-aminolaevulinic acid, porphobilinogen and protoporphyrin IX in plasma

Plasma ALA, PBG, and PPIX kinetics are shown in Figure 2. After the initial absorption and distribution phase the decrease in ALA, PBG and PPIX followed first-order kinetics with half-lives of 1.8, 5.9 and 6.7 h respectively. The range in the half-lives of ALA, PBG and PPIX between different patients was considerable: 1.1–2.5 h for ALA, 4.1–11.6 h for PBG and 2.5–12.8 h for PPIX. In all patients, peak concentrations of ALA were detected at 1 h

**Table 1** Haem biosynthetic enzyme activities before ALA ingestion and the concentrations of PPIX and ‘PPIX equivalents’ (pmol per mg protein) at a mean time interval of 6.7 h after ALA ingestion (60 mg $kg^{-1}$) in gastro-oesophageal tissues of nine patients

| Tissue type | ‘PPIX equivalents’ ALA/8 | ‘PPIX equivalents’ PBG/4 | PPIX | PBG-D activity | FC activity | PDT power index |
|---|---|---|---|---|---|---|
| SQ | 134 ± 38 | 312 ± 59 | 92 ± 15 | 20 ± 3 | 444 ± 49 | 1.0 |
| BE | 132 ± 39 | 201[a] ± 38 | 77 ± 17 | 39[a] ± 6 | 696 ± 89 | 1.4 ± 0.2 |
| AC | 101 ± 38 | 126[a] ± 30 | 112 ± 45 | 39[a] ± 4 | 532 ± 68 | 1.9[a] ± 0.3 |
| GC | 61 ± 19 | 129[a] ± 29 | 57[a] ± 10 | 21 ± 1 | 688[a] ± 38 | 0.7[a] ± 0.1 |

[a]$P$ < 0.05 compared to SQ

**Table 2** Patient and tissue characteristics of nine patients after the oral administration of 60 mg kg$^{-1}$ ALA

| | Age (yrs) | Sex | Tumour diff. grade | Grade of dysplasia | Sampling time (h) | PPIX SQ | PPIX BE | PPIX AC | PPIX GC |
|---|---|---|---|---|---|---|---|---|---|
| *1* | 51 | M | moderately | LGD | 10 | 86 | 46 | 31 | 67 |
| *2* | 45 | M | moderately | ND | 5.25 | 55 | 55 | 82 | 60 |
| *3* | 79 | F | – | HGD | 6.16 | 172 | 173 | | 125 |
| *4* | 73 | M | well | LGD | 6 | 51 | 40 | 78 | 36 |
| *5* | 69 | M | poorly | ND | 7.83 | 85 | 107 | 142 | 54 |
| *6* | 72 | M | poorly | ND | 6 | 80 | 84 | 60 | 44 |
| *7* | 77 | F | poorly | ND | 6.67 | 68 | 24 | 50 | 36 |
| *8* | 63 | M | poorly | LGD | 7.83 | 60 | 34 | 37 | 26 |
| *9* | 45 | M | moderately | LGD | 6 | 168 | 127 | 414 | 64 |

ND = no dysplasia; LGD = low-grade dysplasia; HGD = high-grade dysplasia; Sampling time = sampling time after ALA; PPIX concentrations in pmol per mg protein. Data in bold, selective accumulation of PPIX compared to other tissue samples of the same patient and concentrations declined to baseline levels at 24 h after administration. There was a considerable variability between patients in the time to achieve the peak plasma concentrations of PBG and PPIX (range: 6–12.3 h). Plasma concentrations of PBG and PPIX declined to baseline levels at 48 h after ALA ingestion.

### Side-effects

Side-effects were vomiting, skin photosensitivity, hypotension and transient increases of ASAT and ALAT. Eight patients suffered from at least one of these side-effects. Three patients vomited incidentally between 2.5–4.5 h after the administration of ALA. ASAT and ALAT were elevated 2–3-fold above normal levels in six patients and peaked at day 2 after ALA administration. Mild skin photosensitivity, characterized by itching and mild erythema was present in seven patients. One patient had severe oedema of his facial skin, lips and tongue. The symptoms improved spontaneously within 24 h. Hypotension was found in five patients, in four patients intra-operatively, within 6 h after the administration of ALA. The mean systolic blood pressure of these patients dropped from 125–70 mmHg and the mean diastolic blood pressure from 70–40 mmHg.

### DISCUSSION

In patients with AC in BE we previously found increased activities of PBG-D and FC in endoscopic biopsies of BE and AC compared with SQ (Hinnen et al, 1998). Based on this observation we proposed a PDT power index, the ratio between PBG-D and FC activity in BE and AC in comparison with SQ. We suggested that this index could be of value in predicting porphyrin concentrations in these tissues after ALA administration.

Under the conditions chosen in the present study, however, the PDT power index did not predict the level of intracellular PPIX accumulation found at a mean time interval of 6.7 h after ALA administration in BE, AC, SQ and GC in these patients. It is possible that a relationship still exists between the PDT power index and PPIX accumulation at another time-interval as we found higher enzyme activities and lower PPIX precursor concentrations in AC and BE compared to SQ (Table 1). It is therefore possible that PPIX levels could have peaked at earlier time intervals in BE and AC compared to SQ.

As found by others, who determined PPIX by fluorescence microscopy, an indirect, semi-quantitative method (Regula et al,

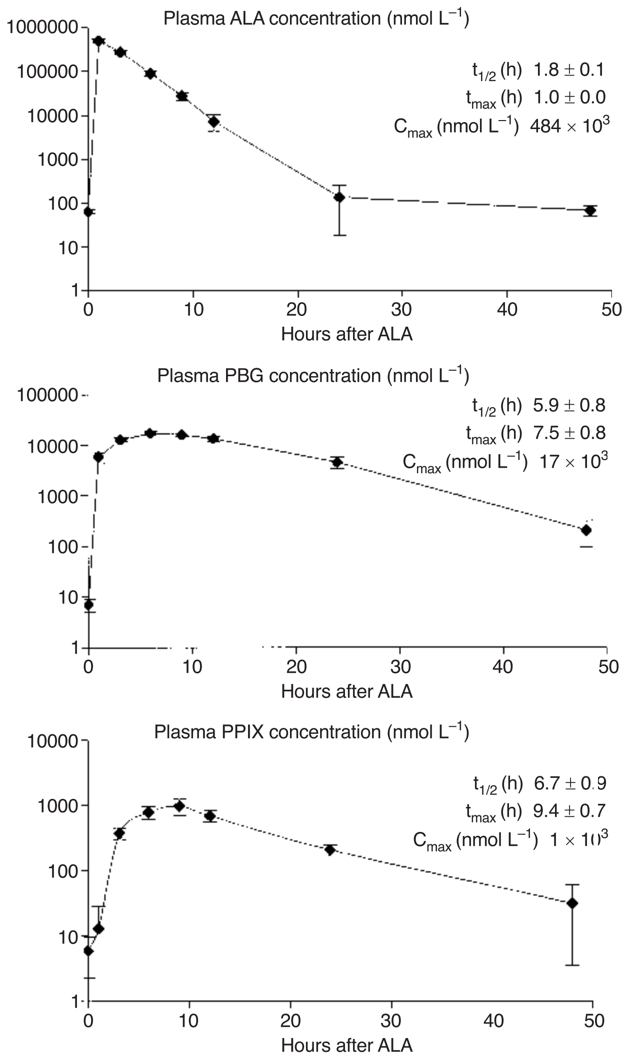

**Figure 2** Pharmacokinetics of ALA, PBG and PPIX in plasma of 10 patients after ingestion of ALA (60 mg kg$^{-1}$). The plasma concentrations are expressed as log-values (y-axis, means ± SEM)

1995; Barr et al, 1996), there seemed to be little selectivity of PPIX accumulation in BE. In contrast, we found selective accumulation of PPIX in four of eight cases of AC. The other four AC samples contained only low levels of PPIX, as compared to SQ. In

 *© 2000 Cancer Research Campaign*

three of these cases the histology showed a poorly differentiated tumour. It has been reported that the grade of differentiation can have a negative or positive effect on the ability of cells to accumulate porphyrins, depending on the type of tissue (Li et al, 1999). In the fourth patient without selectivity between AC and SQ, tissue was collected at a rather late time (10 h) after the administration of ALA, at which time PPIX could already have been converted into haem.

Not only is the absolute intracellular PPIX concentration an important factor for the effect of PDT but also the intracellular localization of PPIX at the time of application of PDT, the duration of illumination and the flux of PPIX in cells (Iinuma et al, 1994; Hinnen et al, 2000b). If oxygen levels are high enough, more PPIX molecules per time-unit result in a greater oxygen radical yield, and therefore will have a more pronounced effect (Henderson and Dougherty, 1992).

The rapid kinetics of ALA and PPIX found in plasma (Figure 2) explain why ALA is an attractive pro-drug for PDT. PPIX in plasma is derived from liver and other cells and the decline in plasma levels reflects a decline in tissue levels (van den Boogert et al, 1998). Because of this rapid decline in PPIX levels, skin photosensitivity is only short-lasting (Barr et al, 1996; Gossner et al, 1998).

A severe side-effect observed in this study was hypotension. Herman et al, recently studied the haemodynamic effects of ALA. A relevant observation in that study was a significant decrease in the systolic and diastolic blood pressure in all six patients (Herman et al, 1998). Goldberg et al found in animal studies evidence for an ALA-triggered histamine release, which could result in vasodilatation and in that way hypotension (Goldberg and McGillion, 1973). Based on these findings we treated four of our patients with antihistaminic agents and corticosteroids prior to ALA administration, but this failed to prevent hypotension in all of them. Haemodynamic stability was restored by infusion of isotonic fluids and plasma. It is presently not clear whether ALA, PPIX or a metabolite is responsible for this side-effect.

In conclusion, this study describes the photodynamic potential of the haem biosynthetic pathway in tissues of patients with BE and AC. At a mean time-interval of 6.7 h after ALA administration, PPIX accumulation could not be predicted from the PDT power index. Selectivity of PPIX accumulation was found in half of the cases of AC but not in BE. The optimum time interval is still not established but is possibly found at an earlier time interval after ALA administration. Side-effects after ingestion of 60 mg $kg^{-1}$ ALA can be serious and a hypotensive response can occur. Optimizing the results of ALA-PDT in the treatment of BE and AC requires further effort in studies concerning the kinetics of ALA and its products in target tissues.

### AKNOWLEDGEMENTS

This study was supported by a grant from ASTRA Zeneca, The Netherlands.

### REFERENCES

Barr H, Shepherd NA, Dix A, Roberts DJ, Tan WC and Krasner N (1996) Eradication of high-grade dysplasia in columnar-lined (Barrett's) oesophagus by photodynamic therapy with endogenously generated protoporphyrin IX. *Lancet* **348:** 584–585

Bishop DF and Desnick RJ (1982) Assays of the heme biosynthetic enzymes. Preface. *Enzyme* **28:** 91–93

Cameron AJ and Carpenter HA (1997) Barrett's esophagus, high-grade dysplasia, and early adenocarcinoma: a pathological study. *Am J Gastroenterol* **92:** 586–591

Clark GW, Ireland AP and DeMeester TR (1996). Dysplasia in Barrett's esophagus: diagnosis, surveillance and treatment. *Dig Dis* **14:** 213–227

Drewitz DJ, Sampliner RE and Garewal HS (1997) The incidence of adenocarcinoma in Barrett's esophagus: a prospective study of 170 patients followed 4.8 years. *Am J Gastroenterol* **92:** 212–215

Edwards MJ, Gable DR, Lentsch AB and Richardson JD (1996) The rationale for esophagectomy as the optimal therapy for Barrett's esophagus with high-grade dysplasia. *Ann Surg* **223:** 585–589

Goldberg A and McGillion FB (1973) Proceedings: central uptake and cardiovascular effects of delta-aminolaevulinic acid. *Br J Pharmacol* **49:** 178P

Gossner L, Stolte M, Sroka R, Rick K, May A, Hahn EG and Ell C (1998) Photodynamic ablation of high-grade dysplasia and early cancer in Barrett's esophagus by means of 5-aminolevulinic acid. *Gastroenterology* **114:** 448–455

Haggitt RC (1994) Barrett's esophagus, dysplasia, and adenocarcinoma. *Hum Pathol* **25:** 982–993

Hameeteman W, Tytgat GN, Houthoff HJ and van den Tweel JG (1989) Barrett's esophagus: development of dysplasia and adenocarcinoma. *Gastroenterology* **96:** 1249–1256

Henderson BW and Dougherty TJ (1992) How does photodynamic therapy work? *Photochem Photobiol* **55:** 145–157

Herman MA, Webber J, Fromm D and Kessel D (1998) Hemodynamic effects of 5-aminolevulinic acid in humans. *J Photochem Photobiol B* **43:** 61–65

Hinnen P, de Rooij FWM, Velthuysen van MLF, Edixhoven A, Hillegersberg van R, Tilanus HW, Wilson JHP and Siersema PD (1998) Biochemical basis of 5-aminolaevulinic acid-induced protoporphyrin IX accumulation: a study in patients with (pre)malignant lesions of the esophagus. *Br J Cancer* **78:** 679–682

Hinnen P, de Rooij FWM, Voortman G, Tilanus HW, Wilson JHP and Siersema PD (2000*a*) Acrylate yellow filters in operating lights protect against photosensitization tissue damage. *Br J Surg* **87:** 231–235

Hinnen P, Siersema PD, Edixhoven A, Wilson JHP and de Rooij FWM (2000*b*) Ferrochelatase activity inhibition by 5-aminolaevulinic acid-induced photodynamic therapy. *submitted for publication*

inuma S, Farshi SS, Ortel B and Hasan T (1994) A mechanistic study of cellular photodestruction with 5-aminolaevulinic acid-induced porphyrin. *Br J Cancer* **70:** 21–28

Kennedy JC and Pottier RH (1992) Endogenous protoporphyrin IX, a clinically useful photosensitizer for photodynamic therapy. *J Photochem Photobiol B* **14:** 275–292

Li G, Szewczuk MR, Pottier RH and Kennedy JC (1999) Effect of mammalian cell differentiation on response to exogenous 5-aminolevulinic acid. *Photochem Photobiol* **69:** 231–235

Lowry O, Rosebrough N, Farr A and Randall R (1951) Protein measurement with the Folin phenol reagent. *J. Biol. Chem.* **193:** 265–275

Regula J, MacRobert AJ, Gorchein A, Buonaccorsi GA, Thorpe SM, Spencer GM, Hatfield AR and Bown SG (1995) Photosensitisation and photodynamic therapy of oesophageal, duodenal, and colorectal tumours using 5 aminolaevulinic acid induced protoporphyrin IX – a pilot study. *Gut* **36:** 67–75

van den Boogert J, van Hillegersberg R, de Rooij FW, de Bruin RW, Edixhoven-Bosdijk A, Houtsmuller AB, Siersema PD, Wilson JH and Tilanus HW (1998) 5-Aminolaevulinic acid-induced protoporphyrin IX accumulation in tissues: pharmacokinetics after oral or intravenous administration. *J Photochem Photobiol B* **44:** 29–38

van der Burgh A, Dees J, Hop WC and van Blankenstein M (1996) Oesophageal cancer is an uncommon cause of death in patients with Barrett's oesophagus. *Gut* **39:** 5–8

van Hillegersberg R, van den Berg JW, Kort WJ, Terpstra OT and Wilson JH (1992) Selective accumulation of endogenously produced porphyrins in a liver metastasis model in rats. *Gastroenterology* **103:** 647–651
